# CPT1A/2-Mediated FAO Enhancement—A Metabolic Target in Radioresistant Breast Cancer

**DOI:** 10.3389/fonc.2019.01201

**Published:** 2019-11-15

**Authors:** Shujun Han, Ryan Wei, Xiaodi Zhang, Nian Jiang, Ming Fan, Jie Hunter Huang, Bowen Xie, Lu Zhang, Weili Miao, Ashley Chen-Ping Butler, Matthew A. Coleman, Andrew T. Vaughan, Yinsheng Wang, Hong-Wu Chen, Jiankang Liu, Jian Jian Li

**Affiliations:** ^1^Department of Radiation Oncology, School of Medicine, University of California, Davis, Sacramento, CA, United States; ^2^Center for Mitochondrial Biology and Medicine, Key Laboratory of Biomedical Information Engineering of Ministry of Education, School of Life Science and Technology and Frontier Institute of Science and Technology, Xi'an Jiaotong University, Xi'an, Shaanxi, China; ^3^Lewis Katz School of Medicine/St. Luke's University Regional Campus, Temple University, Philadelphia, PA, United States; ^4^Department of Chemistry, University of California, Riverside, Riverside, CA, United States; ^5^NCI-Designated Compressive Cancer Center, School of Medicine, University of California, Davis, Sacramento, CA, United States; ^6^Department of Biochemistry and Molecular Medicine, University of California, Davis, Sacramento, CA, United States

**Keywords:** breast cancer stem cells, CPT1A/CPT2, FAO, metabolism, radioresistance, breast cancer

## Abstract

Tumor cells, including cancer stem cells (CSCs) resistant to radio- and chemotherapy, must enhance metabolism to meet the extra energy demands to repair and survive such genotoxic conditions. However, such stress-induced adaptive metabolic alterations, especially in cancer cells that survive radiotherapy, remain unresolved. In this study, we found that CPT1 (Carnitine palmitoyl transferase I) and CPT2 (Carnitine palmitoyl transferase II), a pair of rate-limiting enzymes for mitochondrial fatty acid transportation, play a critical role in increasing fatty acid oxidation (FAO) required for the cellular fuel demands in radioresistant breast cancer cells (RBCs) and radiation-derived breast cancer stem cells (RD-BCSCs). Enhanced CPT1A/CPT2 expression was detected in the recurrent human breast cancers and associated with a worse prognosis in breast cancer patients. Blocking FAO via a FAO inhibitor or by CRISPR-mediated CPT1A/CPT2 gene deficiency inhibited radiation-induced ERK activation and aggressive growth and radioresistance of RBCs and RD-BCSCs. These results revealed that switching to FAO contributes to radiation-induced mitochondrial energy metabolism, and CPT1A/CPT2 is a potential metabolic target in cancer radiotherapy.

## Introduction

Radiation therapy (RT) is the major modality in treatment of solid cancer, including breast cancer (BC), with reported clinical benefits (1, 2). A meta-analysis of 10,801 women with or without RT after breast-conserving surgery in randomized trials demonstrated that RT reduced the 10-year risk of any first recurrence (locoregional or distant tumors) from 35.0 to 19.3% and reduced the 15-year risk of BC mortality rate from 25.2 to 21.4% (1). Cancer stem cells (CSCs; also termed as tumor-initiating cells, TICs) are suggested to be the carcinogenic cell source and responsible for tumor aggressive phenotype and failure of anti-tumor therapy (3, 4). To further improve the BC long-term efficacy by RT, the mechanisms linked with the adaptive radioresistance and recurrent risk in CSCs are to be investigated (5–8).

Elucidation of the metabolic dynamics of resistant breast cancer cells (RBCs) will help to identify metabolic targets to synergize the efficacy of RT. The theory of aerobic glycolysis in malignant cells (Warburg Effect) (9–11) is being updated, with emerging new evidence indicating an adaptive energy reprogramming in tumor cells with mitochondrial reactivation and oxidative phosphorylation (12, 13). We have reported that mitochondrial MnSOD activity is required for radioresistance in BC cells (14, 15) and radiation can enhance mitochondrial OXPHOS in tumor cells (16). Enhanced mitochondrial bioenergetics are found to be the major cellular fuel supplement for cell cycle progression (17–19), tumor aggressive phenotype and metastasis (20). Accumulating new evidence indicates that reprogramming in mitochondrial metabolism is actively involved in tumor proliferation and metastasis (12, 21–23). Mitochondrial energy output is also required for nuclear DNA repair after IR (24), and mitochondrial FAO is linked with BC metastasis (25, 26). With such a flexible adaptive energy metabolism detected in the malignant cells (13, 27), it is reasonable to look into the deep mechanistic insights of reprogramming mitochondrial bioenergetics in aggressive tumors, especially the RBCs.

Under genotoxic stress conditions, such as chemotherapy or radiotherapy, tumor cells must acquire additional cellular fuel resource to meet the increased demands of energy consumption for damage repair and survive (24). In addition to glucose, fatty acids with high ATP yield are a relevant energy-rich source in cancer cells under such genotoxic crisis. The FAO-mediated mitochondrial bioenergetics has been assumed to play a critical role in cell proliferation, cancer stemness and chemoresistance (28–30). Inhibition of mitochondrial FAO by etomoxir impairs NADPH production and increases reactive oxygen species (ROS), resulting in ATP depletion and cell death in human glioblastoma cells (31). In mitochondrial FAO pathways, targeting CPT1A generates clinic benefits in RT for nasopharyngeal carcinoma patients (32). However, the precise network of FAO enhancement in reprogramming mitochondrial energy metabolism, especially in resistant breast cancer cells, remains to be elucidated.

To mimic clinical radioresistance, in this study, RBCs were generated from wild type breast cancer cells via continuous ironizing radiation (IR), and the radioresistant BCSCs cells were sorted from the RBCs (8), termed as radiation-derived BCSCs (RD-BCSCs). Using proteomics of RD-BCSCs and CRISPR-mediated FAO gene editing, here we revealed a novel mitochondrial lipid metabolic reprogramming in RBCs and RD-BCSCs. The mitochondrial fatty acid oxidation (FAO) was enhanced in both RBCs and RD-BCSCs and linked with recurrent BC and a worse prognosis in BC patients. Blocking FAO by CRISPR-mediated CPT1A/CPT2 KO inhibited aggressive phenotype of the radioresistant BC with down-regulation of the ERK pathway, indicating a potential metabolic target in breast cancer radiotherapy.

## Materials and Methods

### RBCs, RD-BCSCs, and Other Reagents

MCF7 and MDA-MB-231 human breast cancer cells were purchased from ATCC (Manassas, VA, USA). MCF7/C6 radioresistant clone is a single clone surviving from MCF7/WT cells after fractionated ionizing radiation treatment (2 Gy × 15) (33, 34). MDA-MB-231/C4 are radioresistant breast cancer cells surviving from MDA-MB-231/WT cells after fractionated radiation (2 Gy × 30) (35). MCF7, MCF7/C6, MDA-MB-231, and MDA-MB-231/C4 cells were cultured in DMEM medium with 10% fetal bovine serum (FBS, Life Technologies, NY, USA) and 1% penicillin/streptomycin (Life Technologies). RD-BCSCs were sorted as previously described (8, 36). Cell pellets of MCF7/C6 were rinsed with cold PBS with 2% FBS and then suspended with PBS containing 0.5% FBS and 0.5 mg/mL PI (Sigma, St. Louis, MO, USA). Then, the cell suspension was sorted using Cytopeia influx Cell Sorter (BD Biosciences, San Jose, CA, USA). The antibody against HER2/neu was conjugated to allophycocyanin (APC, BD Biosciences, San Jose, CA), anti-human CD44 was conjugated to FITC, and human CD24 was conjugated to phycoerythrin (PE; Invitrogen, Carlsbad, CA). Cell viability was assessed by 7-AA staining during cell sorting and then determined by trypan blue exclusion after sorting (Figure S1A). The isolated RD-BCSCs (CD44^+^/CD24^−/low^/HER2^+^) were maintained in CSC medium containing free serum and supplemented with B27 (Life Technology, Carlsbad, CA, USA), 20 ng/ml EGF (Biovision, Mountain View, CA, USA), 20 ng/ml basic-FGF, and 4 μg/ml heparin (VWR, Philadelphia, PA, USA). Cells were cultured in ultralow-attachment Petri dishes with 5% CO_2_ at 37°C. All cell lines were tested mycoplasma free before experiments. Etomoxir was purchased from Sigma-Aldrich. Oil Red O was obtained from Millipore Sigma (St. Louis, MO, USA).

### Western Blot

Western blot was performed as previously described (37). Briefly, the cell lysates were separated on SDS-PAGE gels and transferred to PVDF membranes. The membranes were blocked with 5% milk for 1 h, and then incubated with primary antibodies with shaking at 4°C overnight. In the next day, the membranes were incubated with horseradish peroxidase-conjugated secondary antibodies for 1 h at room temperature. The protein blots were developed using an enhanced chemiluminescence western blot detection system (BioRad, Hercules, CA, USA). Antibodies against HER2/Neu (C-18, #SC284), CPT2 (G-5, #SC-377294), HADHA (E-8, #SC374497), HADHB (E-1, #SC-271495), ERK1/2 (C-9, #SC514302), and c-Fos (E-8, SC#166940) were all diluted at 1:500 and purchased from Santa Cruz Biotechnology (Santa Cruz, CA, USA). Anti-CPT1A (clone D3B3, #12252), Phospho-p44/42 MAPK (Thr202/Tyr204, clone 197G2, #4377), Phospho-GSK3 β (Ser9, #9336), GSK3 β (clone 27C10, #9315), Phospho-STAT3 (Ser727, clone D8C2Z, #94994), and Phospho-JNK (Thr183/Tyr185, #9251) were from Cell Signaling Technology (Beverly, MA, USA). Antibody against ACAD9 (#3170340688) was purchased from Novus Biologicals (Littleton, CO, USA). Anti-β-actin at 1:2000 (#A2066) was from Sigma-Aldrich.

### Fatty Acid Oxidation (FAO) Assay

FAO measurement was following the manufacturer's instruction of Fatty Acid Oxidation Assay Kits from Abcam (ab217602). Briefly, 3 × 10^4^ cells were seeded per well in 96-well plates and cultured overnight. Then the cells were rinsed twice with 100 μl prewarmed FA-Free medium followed by adding 90 μL pre-warmed FA Measurement Medium. The wells without cells were used as signal control. A total of 85 μL of FA-Free Measurement medium was added to the wells, and 5 μL of BSA control were included as the FA-free control. All wells except the blank control had 10 μL Extracellular O_2_ Consumption Reagent added. The FAO activator FCCP (0.625 μM) and inhibitor Etomoxir (40 μM) were added as the positive and negative controls. Then the wells were sealed with 100 μL pre-warmed mineral oil, and the FAO was measured using the condition as Extracellular Oxygen Consumption. The results were normalized by the protein concentration with the cells in each sample under the BCA assay.

### Lipid Accumulation Assay

Breast cancer cells were plated and cultured in 96-well plates. In the next day, cells were treated with 250 μM free fatty acid (oleic acid: palmitate acid = 2:1) for 48 h. After being washed with PBS twice, cells were fixed with 4% paraformaldehyde under room temperature (RT) for 30 min. Cells were washed with sterilized water once and added into 50 μL Oil Red working solution; they were then incubated for 15 min at RT. Then 50 μL 60% isopropanol was added to the cells for 20 s at RT. Finally, the cells were washed with water twice, and the images were obtained using a Nikon microscope (Eclipse, E1000M, Japan). The red oil dye was eluted with 50 μL DMSO and incubated for 10 min with gentle shaking. The lipid accumulation results were determined by the fluorescence microplate spectrophotometer (Molecular Devices) at 510 nm.

### Oxygen Consumption

Extracellular Oxygen Consumption detection was performed following the instruction of kit from Abcam (ab197243,) with 3 × 10^4^ cells seeded per well in 96-well plates and cultured at 37°C overnight. The cell medium was replaced with 150 μl fresh culture media followed by adding 10 μl of extracellular oxygen consumption reagent. The wells without oxygen consumption reagent were used as blank control. Wells with Etomoxir (40 μM) added were included as the negative control. Wells had 100 μl of pre-warmed mineral oil added to avoid the air bubbles. The plates were read immediately in a fluorescence microplate spectrophotometer (Molecular Devices) at 37°C. The signals were collected at 1.5 min intervals for 90–120 min at Ex/Em = 380/650 nm. The results were normalized by protein concentration of the cells in each sample under the BCA assay.

### MTT Assay

Breast cancer cells were seeded in 96-well plates for 24 h. After they proliferated to about 90% confluence, the cells were added into 50~100 μL MTT solution (M-2128, Sigma) and cultured at 37°C for 2 h. The results were measured in a microplate spectrophotometer (Molecular Devices) at 540 nm.

### Cell Apoptosis Assay

Breast cancer cells were rinsed by cold PBS twice, collected and stained using the Annexin-V/PI kit (Biosource, Invitrogen, Carlsbad, CA, USA) according to manufacturer's protocol. Stained cells were analyzed by flow cytometry (Becton Dickinson canto II, BD, NJ, USA). Data were analyzed using Flowjo software (Three Star, Inc., Ashland, OR, USA).

### Colony Formation Assay

For measuring cellular clonogenicity, 1 × 10^3^ cells were seeded into 6-well plates and treated with or without 5 Gy radiation in the next day. Cells were then cultured for 14 days at 37°C. The colonies were fixed and stained with Coomassie blue, and then colony formation rate was determined by counting colonies in each group. Finally, the colony images were observed and recorded by a Nikon microscope (Eclipse, E1000M, Japan) (8).

### Immunohistochemical Staining (IHC)

The slides of primary biopsy tissues and recurrent tumors from patients with breast cancer were tested by immunohistochemistry (IHC) using Vectastain ABC kit and DAB Peroxidase Substrate kit SK-4100 (Vector Laboratories, Burlingame, CA, USA). The prepared tumor slides were firstly deparaffinized, hydrated, and then covered with blocking buffer for 1 h after heat-induced epitope retrieval. The slides were incubated with anti-CPT1A (Cell Signaling Technology, #12252) and anti-CPT2 (Santa Cruz, sc-20671) at 1:200 at 4°C overnight, followed by washing with PBST three times, and then incubated with the secondary antibody for 30 min at RT. The slides were then covered with ABC solution for 30 min on the shaker at RT. The slides were incubated with DAB solution about 2 min and then transferred to hematoxylin, HCl solution and Li_2_CO_3_ solution quickly several times. Finally, the slides were dehydrated and sealed. The slides were observed and recorded by Nikon microscope (Eclipse, E1000M).

### CRISPR-Mediated Gene Editing

The single guide RNA (sgRNA) was designed according the CRISPR Design in Zhang Lab https://zlab.bio/guide-design-resources. We created oligonucleotide to target genes CPT1A and CPT2. The sgRNA sequences are designed as follows: human CPT1A: CTCCGGACGGGATTGACCTG; human CPT2: CGGGGCCCCGCGGTTGGTCC. The LentiCRISPRv2 backbone was used, which contains the hSpCas9 and sgRNA expression cassettes. Plasmids were purchased from the Addgene plasmid repository (Addgene #52961) (https://www.addgene.org/). Backbone LentiCRISPRv2 was annealed to oligonucleotides following the Zhang Lab GeCKO protocol and packaged into lentiviruses. The Lentiviral particles were produced in HEK293T cells following the protocol from Addgene (38), and breast cancer cells were infected with lentiviruses and selected with 0.3 μg/ml puromycin. Western blot analysis was performed to identify cell colonies with gene deficiency.

### Proteomics of RD-BCSCs and MCF7 Cells

The protein mixture from total cell lysates of RD-BCSCs and MCF7 was first treated with dithiothreitol and iodoacetamide for cysteine reduction and alkylation, respectively. The protein samples were then digested using modified sequencing-grade trypsin (Roche, Basel, Switzerland) at an enzyme/substrate ratio of 1:100 in 50 mM NH_4_HCO_3_ (pH 8.5) at 37°C overnight. The peptide mixture was subsequently dried in a Speed-vacuum and desalted by employing OMIX C_18_ pipet tips (Agilent Technologies, Santa Clara, CA, USA), reconstituted in water and subjected to LC-MS and MS/MS analyses on a Q Exactive Plus mass spectrometer equipped with a nanoelectrospray ionization source. Samples were automatically loaded from a 48-well microplate autosampler using an EASY-nLC 1200 system (Thermo Fisher Scientific, Rockford, IL, USA) at 3 μL/min onto a biphasic precolumn (150 μm i.d.) comprised of a 3.5-cm column packed with 5 μm C_18_ 120 Å reversed-phase material (ReproSil-Pur 120 C_18_-AQ, Dr. Maisch). The biphasic trapping column was connected to a 20-cm fused-silica analytical column (PicoTip Emitter, New Objective, 75 μm i.d.) with 3 μm C_18_ beads (ReproSil-Pur 120 C_18_-AQ, Dr. Maisch). The peptides were then separated using a 180-min linear gradient of 2–45% acetonitrile in 0.1% formic acid and at a flow rate on 250 nL/min. The mass spectrometer was operated in a data-dependent scan mode. Full-scan mass spectra were acquired in the range of *m/z* 350–1,500 using the Orbitrap analyzer with a resolution of 70,000. Up to 25 of most abundant ions found in MS with a charge state of 2 or above were sequentially isolated and collisionally activated in the HCD cell with collision energy of 27 to yield MS/MS.

### Bioinformatics Analysis

Maxquant (Version 1.5.2.8) was used to analyze the LC-MS and MS/MS data for the identification and quantification of proteins in the LFQ mode (39). The maximum number of mis-cleavages for trypsin was two per peptide. Cysteine carbamidomethylation was set as a fixed modification. Methionine oxidation and phosphorylation on serine, threonine, and tyrosine were set as variable modifications. The tolerances in mass accuracy for MS and MS/MS were both 20 ppm. Maximum false discovery rates (FDRs) were set to 0.01 at both peptide and protein levels, and minimum required peptide length was six amino acids. The LC-MS and MS/MS protein data were also analyzed with functional clustering. Of all proteins in our total protein array, only proteins that showed levels of detection were submitted to DAVID Bioinformatics Resources v6.7 (https://david.ncifcrf.gov/). Parameters were established for our functions of interest with a cutoff of *p* < 0.05.

### Category Selection of Proteomics

DAVID Bioinformatics Resources provide a wealth of information within the Gene Ontology Tool for Biological Function (40, 41). Different broad categories were generated to profile the cluster of proteins related to varied cellular functions including mitochondrial bioenergetics and lipid metabolism as well as FAO. We used the Uniprotein tagging system, UPKeyword due to the high number of hits in the protein list.

### Tumorsphere Formation

Tumorsphere assay was performed as described (42). Cells were sieved with 40 μm cell strainers (Fisher, Failawn, NJ, USA) and single-cell suspensions were seeded into 60 mm Petri dishes. The cells were grown in serum-free mammary epithelial basal medium (MEBM, Lonza, Walkersville, MD, USA), supplemented with B27, 20 ng/ml EGF, 20 ng/ml basic-FGF, and 4 μg/ml heparin. Cells were then cultured for 10 days, and tumor spheres were counted under light microscopy.

### Three-Dimensional (3D) Morphogenesis Assay

MCF7 and RD-BCSC cells in 40 μL plug of Matrigel (growth factor reduced and phenol red free, Becton Dickinson, Plymouth, UK) were plated to the well of an 8-well LabTek Chambered coverglass (Nunc, Rochester, USA) at 37°C for 30 min. On ice, cells were prepared at a concentration of 5,000 cells/ml in KSFM supplemented with 5 ng/ml EGF, 2% (v/v) FCS, 4% (v/v) Matrigel, and 0.2 mL of this cell solution was plated onto the Matrigel plug and incubated for 30 min at 37°C, after which 0.2 mL of growth medium was added (KSFM supplemented with 5 ng/mL EGF, 2% (v/v) FCS). Culture medium was changed every 2–3 days. At day 10, morphology was assessed by phase microscopy and cells were fixed and processed for immunofluorescence microscopy analysis.

### Statistical Analysis

Statistical significance of differences was evaluated using two-tailed student *t*-test for two groups' comparison or one-way ANOVA test where multiple groups were involved. *p* < 0.05 was considered statistically significant.

## Results

### Mitochondrial FAO Is Enhanced in Radioresistant Breast Cancer Cells

**Figure 1:**
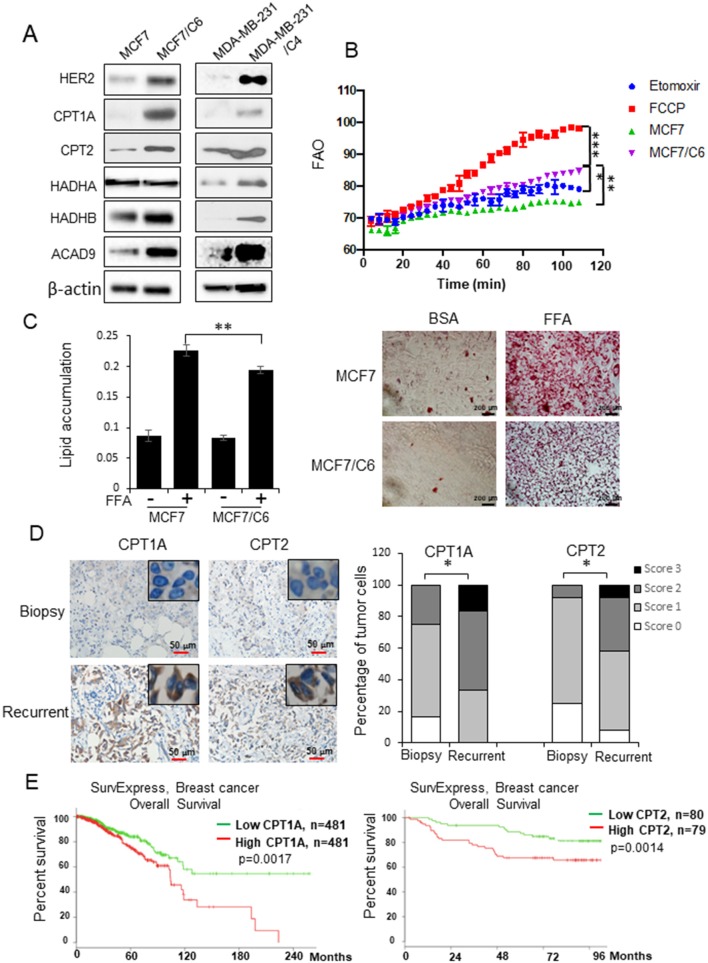
FAO is enhanced in radioresistant BC cells and recurrent BC and linked with a poor prognosis in BC patients. **(A)** Western blot of a cluster of FAO enzymes and HER2 in wild type MCF7, MDA-MB-231, and their counterpart radioresistant MCF7/C6 and MDA-MB-231/C4 cells. **(B)** FAO activity assay of MCF7 and MCF7/C6 cells with MCF7/C6 treated with FAO inhibitor Etomoxir (40 μM) as a negative control and FAO enhancer FCCP (1 μM) as a positive control. **(C)** Fatty acid turnover rate in MCF7 and MCF7/C6 cells treated with or without 250 μM Free Fatty Acid (FFA; oleic acid: palmitic acid = 2:1) for 24 h before Oil Red staining (left: quantitation of lipid accumulation; right: representative images of FFA accumulation). **(D)** Representative IHC of CPT1A and CPT2 in biopsy and recurrent BC (left). Quantitation of IHC was achieved by scoring staining intensity and positive cells (right). **(E)** Elevated CPT1A/CPT2 expression correlates a worse overall survival of BC patients in the TCGA database http://bioinformatica.mty.itesm.mx:8080/Biomatec/SurvivaX.jsp. Error bars in **(B–D)** represent the mean ± standard deviation. Significance was determined by one-way ANOVA test, ^*^p < 0.05, ^**^p < 0.01, ^***^p < 0.001.

Lipid metabolism has been linked with cancer therapy response (28–30). Here we address the question of whether reprogramming mitochondrial FAO plays a key role in breast cancer radioresistance. Two radioresistant BC (RBC) cell lines (MCF7/C6 and MDA-MB-231/C4) isolated from surviving MCF7 and MDA-MB-231 residues with HER2 induction and aggressive phenotype after chronical radiation (Figure S1A) (8, 35) showed enhanced expressions of mitochondrial FAO genes CPT1A, CPT2, HADHB, and ACAD9 (Figure 1A). We then measured the mitochondrial FAO activity by ^18^C-labeled unsaturated fatty acid oleate as the substrates with CPT1A specific inhibitor Etomoxir (ETX) and the FAO activator FCCP as negative and positive controls, respectively, demonstrating a significant elevation of FAO activity in MCF7/C6 vs. wild type MCF7 cells (Figure 1B). Furthermore, enhanced lipid turnover rate was detected in MCF7/C6 cells loaded with free fatty acid (FFA), which was contrasted with the markedly accumulation of FFA in the wild type MCF7 cells (Figure 1C), indicating enhanced FAO metabolism in RBC cells.

We next determined whether the FAO is enhanced in the human recurrent breast tumors compared to the primary tumors. CPT1 and CPT2 are the rate-limiting transporters and play a key role in mitochondrial long-chain FAO and lipid metabolism. Remarkably, the enhanced co-expression of CPT1A (an isoform of CPT1) and CPT2 was mostly detected in the pathological sections of recurrent BC tumors compared to the paired original biopsy specimens in a group of 12 BC patients (Figure 1D). In agreement, the TCGA database revealed a poor prognosis in BC patients with increased expression of CPT1A or CPT2 (Figure 1E) (http://bioinformatica.mty.itesm.mx:8080/Biomatec/SurvivaX.jsp). Together, these results indicate that reprograming mitochondrial FAO contributes to BC radioresistance and worse prognosis.

### CPT1A/CPT2 Mediated FAO Is Required for Radioresistant Breast Cancer Stem Cells

**Figure 2:**
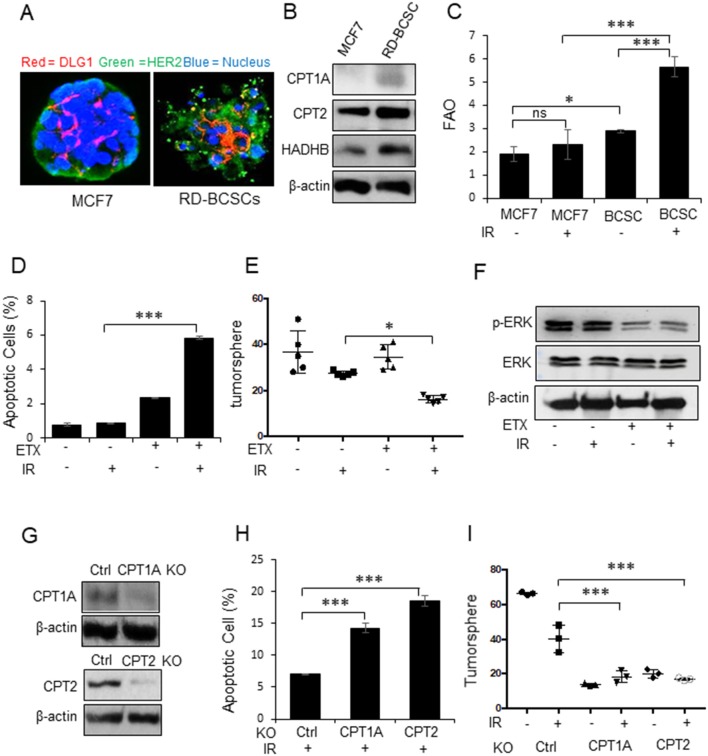
CPT1A/CPT2 mediated FAO is required for radioresistant breast cancer stem cells. **(A)** Representative mammospheres formed from MCF7 and RD-BCSCs assessed by immunofluorescence microscopy (cell polarity protein DLG1, HER2, and nucleus are labeled by red, green, and blue respectively). **(B)** Western blot of FAO enzymes CPT1A, CPT2, and HADHB in MCF7 and RD-BCSCs cells. **(C)** FAO activity in MCF7 and RD-BCSCs with or without IR treatment for 24 h. **(D)** Radiation-induced apoptosis measured in RD-BCSCs with or without FAO inhibition (ETX, Etomoxir 200 μM for 48 h). **(E)** Tumorsphere formation assay of **(D)**. **(F)** Western blot of phosphorylated ERK in RD-BCSCs treated with ETX, IR or combined. **(G)** Western blot of CPT1A and CPT2 in RD-BCSCs with CRISPR-mediated CPT1A and CPT2 KO. **(H)** Radiation induced apoptosis in RD-BCSCs (Control), RD-BCSCs CPT1A KO, and RD-BCSCs CPT2 KO cells. **(I)** Tumorsphere formation of **(H)**. In **(C,D,E,H,I)** *n* = 3; mean ± SD; one-way ANOVA test, ^*^p < 0.05, ^***^p < 0.001.

Our previous results have indicated that mitochondrial energy enhancement is involved in BC aggressiveness due to HER2 expression (15, 43) that is confirmed in Figure 1A. Following the standard biomarkers of breast cancer stem cells (BCSCs) (4) and our established BCSCs from MCF7/C6 with HER2 induction as radiation-derived BCSCs (RD-BCSCs; CD44^+^/CD24^−/low^/HER2^+^ with enhanced ALDH activity (8), we compared the tumorspheres from RD-BCSCs and MCF7 shown in Figure 2A. The tumorsphere of RD-BCSCs showed severely disorganized structure with altered distribution of cellular polarity protein (DLG, red) and enhanced HER2 expression (green), indicating an aggressive phenotype of RD-BCSCs.

Consistent with the results of RBC in Figure 1A, the key FAO enzymes, CPT1A, CPT2, and HADHB, were also enhanced in RD-BCSCs (Figure 2B), and both basal and irradiated FAO levels were elevated in RD-BCSCs whereas no significant FAO elevation was detected in irradiated MCF7 compared to basal level, although the proteomics data showed enhanced mitochondrial proteins in both irradiated both MCF7 and RD-BCSCs (Figure 2C, Figures S2A, S3), suggesting that reprogramming FAO is an unique feature of RD-BCSCs. This is further evidenced by the specifically enhanced cluster of factors in lipid metabolism rather than other cellular proteins in irradiated RD-BCSCs (47 to 81 in RD-BCSCs vs. 55 to 76 in MCF7); and the protein intensity was also more increased in irradiated RD-BCSCs (5.24-fold) contrasted to 2.31-fold in MCF7 (Figures S1B,C). The FAO inhibitor Etomoxir (ETX) significantly enhanced basal and radiation-induced apoptosis with inhibited tumorsphere formation and ERK activity in RD-BCSCs (Figures 2D–F, Figure S2B). In agreement, blocking CPT1A/CPT2 by CRISPR-mediated gene deficiency (Figure 2G), enhanced apoptosis level and reduced the tumorsphere formation in RD-BCSCs upon IR (Figures 2H,I, Figure S2C). Additional proteomics evidence suggested that mitochondrial proteins were more enriched in the RD-BCSCs compared to MCF7 cells after radiation. The total mitochondrial protein counts increased from the same basal 117 in MCF7 and RD-BCSCs to 152 and 163, 29.9, and 39.3% respectively, whereas protein intensity had a 2.64-fold increase (4.50E+10 to 1.06E+11) in RD-BCSCs compared to a 1.83-fold increase in MCF7 cells (Figures S3A,B). Additionally, we further analyzed the mitochondrial proteins and found that FAO metabolism was also demonstrated with an increased protein number, from 11 to 17, in irradiated RD-BCSCs including CPT1A and CPT2 (Figures S4A,B, Table S1), indicating that enhanced mitochondrial FAO contributes to radioresistance of RD-BCSCs.

### ERK Activation Is Linked With Mitochondrial FAO Enhancement

**Figure 3:**
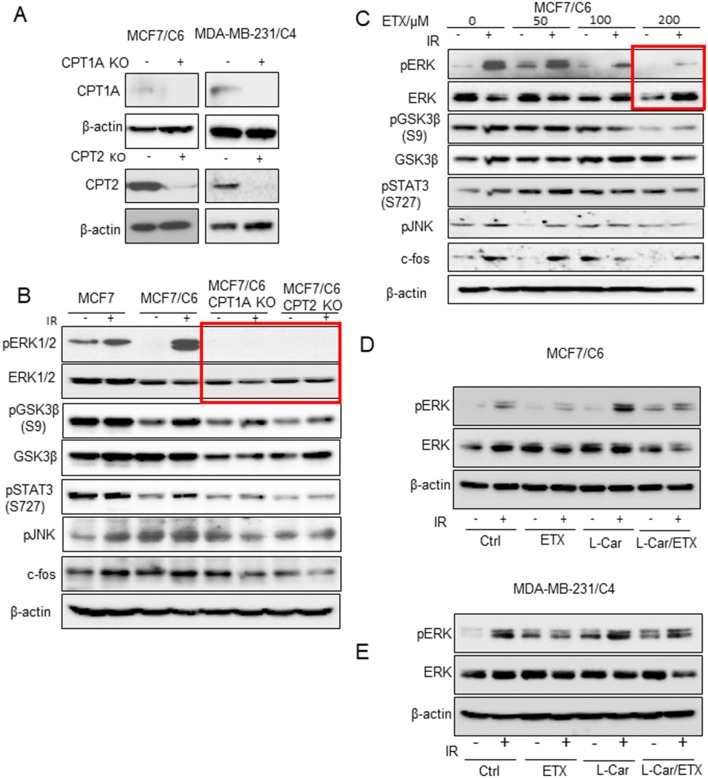
ERK proliferative pathway is linked with FAO enhancement. **(A)** Western blot of CPT1A and CPT2 in control and MCF7/C6 and MDA-MB-231/C4 cells with CRISPR KO CPT1A and CPT2. **(B)** Western blot of proliferative genes in irradiated MCF7, MCF7/C6, MCF7/C6 CPT1A KO, and MCF7/C6 CPT2 KO cells. **(C)** Western blot of MCF7/C6 cells treated with increased doses of ETX for 24 h followed by 5 Gy IR. **(D)** Western blot of pERK in MCF7/C6 cells treated with ETX, L-carnitine (an FAO enhancer) or combined followed by 5 Gy IR. **(E)** Western blot of pERK in MDA-MB-231 cells were treated ETX, L-carnitine (an FAO enhancer) or combined followed by 5 Gy IR.

To explore the key factors responsible for FAO-mediated radioresistance, we tested an array of cell proliferating factors in the two RBC cell lines by CRISPR-mediated knockout of CPT1A and CPT2 (Figure 3A). Strikingly, the activated form of ERK1/2 was absent in the CPT1A and CPT2 KO cells, although other cell growth factors including phosphor-GSK3β, phospho-STAT3, and phospho-JNK were also reduced to a certain degree (Figure 3B). Alternatively, a dose-dependent inhibition of phospho-ERK and its downstream effectors was also determined in MCF7/C6 cells with increasing concentrations of ETX (Figure 3C). It turned out that 200 μm ETX could dramatically block the phosphorylation of ERK1/2. The dependence of ERK1/2 kinase in the FAO-mediated resistance was again evaluated by a rescue experiment, in which the FAO activity was inhibited by ETX and then activated by L-carnitine. As expected, the induction of phospho-ERK1/2 upon radiation was significantly inhibited by etomoxir while enhanced by L-carnitine. Of note, the combination of etomoxir and L-carnitine treatment ablated the phospho-ERK1/2 induction by L-carnitine treatment upon radiation (Figures 3D,E). Together, our inhibition and rescue experiments consistently demonstrated that FAO mediated radioresistance is linked with ERK1/2 activation for the aggressive phenotype of radioresistant breast cancer cells.

### Deficiency of CPT1A or CPT2 Reduces OCR, Cell Viability, and Slowdown Fatty Acid Turnover Rate in RBC Cells

**Figure 4:**
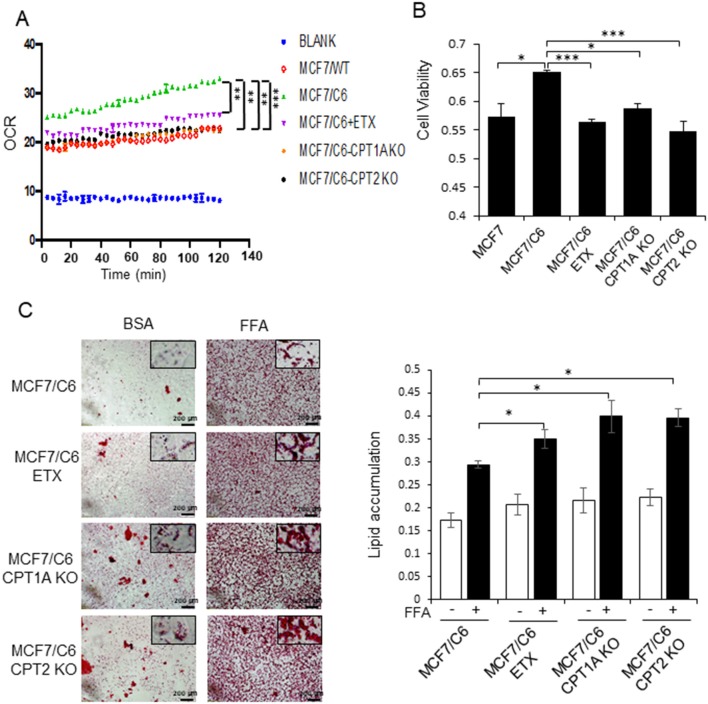
Blocking CPT1A/CPT2 inhibits FAO and fatty acid turnover in RBC cells. **(A)** Basal oxygen consumption rate (OCR) measured in MCF7, MCF7/C6, and MCF7/C6 CPT1A KO and MCF7/C6 CPT2 KO cells with MCF7/C6 cells treated with 40 μM Etomoxir for 48 h as the FAO blockade control. **(B)** Cell viability measured by MTT of **(A)**. **(C)** Fatty acid turnover rate in MCF7/C6, MCF7/C6 CPT1A KO, and MCF7/C6 CPT2 KO cells with MCF7/C6 treated with 40 μM ETX for 48 h as FAO inhibition control; cells were incubated with 250 μM FFA (oleic acid: palmitic acid = 2:1) for an additional 24 h and then Oil Red staining; representative FA turnover rates were shown on the right. In **(A–C)**, *n* = 3; mean ± SD; significance was determined by one-way ANOVA test, ^*^p < 0.05, ^**^p < 0.01, ^***^p < 0.001.

**Figure 5:**
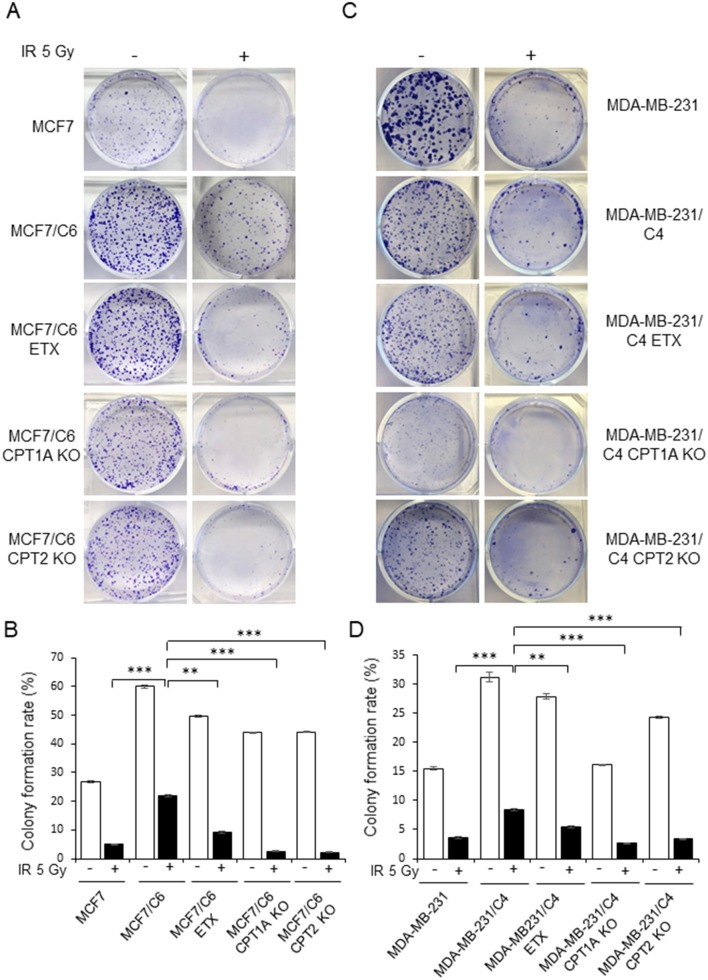
Blocking CPT1A/CPT2 synergizes radiation in eliminating clonogenic RBCs cells. Representative images **(A)** of colonies generated with 1 × 10^3^ MCF7, MCF7/C6, MCF7/C6 CPT1A KO, and MCF7/C6 CPT2 KO cells treated with or without 5 Gy IR, 40 μM ETX, or combined. All colonies were fixed at day 14 and quantitated **(B)**. **(C,D)** Same as **(A,B)** except that MDA231, MDA231/C4 CPT1A KO, MDA231/C4 CPT2 KO, and MDA231/C4 cells were tested. In all experiments, *n* = 3; Error bars in **(B,D)** represent mean ± SD; significance was determined by one-way ANOVA test, ^**^p < 0.01, ^***^p < 0.001.

**Figure 6:**
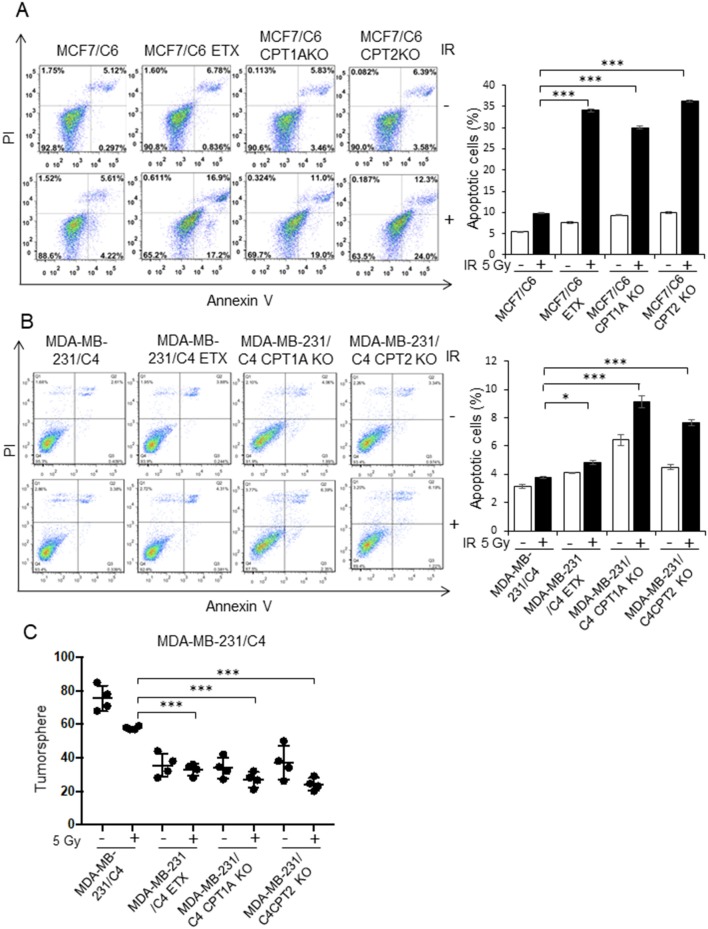
Blocking CPT1A/CPT2 enhances apoptotic cell death and inhibits tumorsphere. **(A)** Representative flow cytometry of MCF7/C6, MCF7/C6 CPT1A KO, MCF7/C6 CPT2 KO, and MCF7/C6 cells treated with 5 Gy IR, ETX (40 μM, 48 h), or combined (left); quantitation of apoptotic cells is shown on the right. **(B)** Repeated experiments with RBC MDA-MB-231/C4 and CRISPR-KO counterparts. **(C)** Tumorsphere assay of **(B)**. In **(A–C)**, *n* = 3; mean ± SD; significance was determined by one-way ANOVA test, ^*^p < 0.05, ^***^p < 0.001.

**Figure 7:**
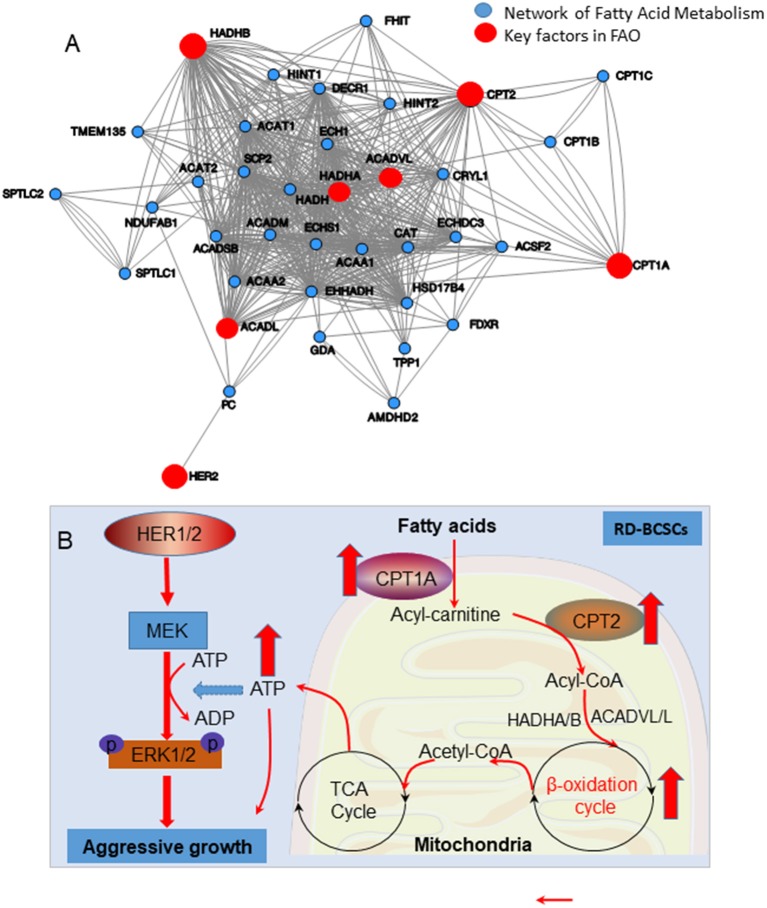
**(A)** The network of enhanced FAO in radioresistant breast cancer cells due to overexpression of mitochondrial FAO and HER2. **(B)** Schematic pathways of HER1/2-MEK-ERK1/2 mediated aggressive growth in radioresistant breast cancer cells due to increased mitochondrial FAO metabolism mediated by CPT1A/CPT2.

By comparing the metabolic features of BC cells and RBCs, we found that both the oxygen consumption rate and cell viability were enhanced in MCF7/C6 cells compared to MCF7 cells, but remarkedly reduced by ETX or in CPT1A/CPT2 KO (Figures 4A,B). Additionally, ETX treatment and CPT1A/CPT2 KO increased FFA accumulation, indicating a slower fatty acid turnover (Figure 4C). The colony formation assay was used to evaluate the cell survival rates when given radiation therapy with FAO inhibition in RBCs. The colonies in the RBC MCF7/C6 and MDA-MB-231/C4 cells were more abundant than the basal clonogenic capacity of the parental MCF7 and MDA-MB-231 cells, indicating an enhanced aggressive phenotype of RBCs. However, the etomoxir treatment and CPT1A/CPT2 KO markedly reduced the survival colony rate in MCF7/C6 and MDA-MB-231/C4 cells (Figures 5A–D). IR induced cellular apoptosis was also evaluated in the RBCs. The FAO inhibition increased radiation-induced apoptosis from 10 to 34% (etomoxir treatment), 31% (CPT1A KO), and 36% (CPT2 KO) in MCF7/C6 cells and from 3.8 to 5% (etomoxir treatment), 9.2% (CPT1A KO), and 7.7% (CPT2 KO) in MDA-MB-231/C4 cells (Figures 6A,B). FAO inhibition also reduced tumorsphere formation from 58 to 34.5 (etomoxir treatment), 26.5 (CPT1A KO), and 24.5 (CPT2 KO) with IR in MDA-MB-231/C4 cells (Figure 6C). Together, these results indicate that CPT1A/CPT2 mediated FAO enhancement is required for the energy demands and cell survival in RBCs. In summary (Figure 7), our results reveal that reprogramming mitochondrial FAO is the major cellular energy supplement in radioresistant breast cancer cells. Such adaptive mitochondrial energy metabolism is linked with the clinical outcome of BC patients treated with radiotherapy. We also reveal that the ERK-mediated prosurvival pathway is a potential downstream target in FAO-mediated aggressive proliferation in BC with enhanced activation of HER2 leading to promoted cell proliferation.

## Discussion

It is highly clinically relevant to reveal the major cellular energy driving the growth of therapy-resistant cancer cells. The concept of aerobic glycolysis in cancer metabolism (Warburg Effect), believing the deficient mitochondrial function in cancer cells, has been updated with accumulating results of active tumor metabolic to oncogenic (9) and genotoxic stress including ionizing radiation (16, 44, 45). A dynamic metabolic feature is linked to the adaptive reprogramming in energy supply, which is required to meet the increased cellular fuel demands for cancer cells to repair the damage and survive anti-cancer therapy. Identification of the metabolic targets required for tumor cell survival under genotoxic stress conditions such as chemo-radiotherapy are necessary for improving the therapeutic efficacy. Among other hallmarks of cancer cell progression, cancer cells can adjust energy metabolism to meet the demands of cellular fuel consumption for proliferation and survival to therapeutic stress conditions (11). In this study, we revealed a unique metabolic feature in RBCs and radioresistant breast cancer stem cells (RD-BCSCs). It showed that FAO enzyme expression and mitochondrial FAO activity were enhanced in response to radiation compared to wild type breast cancer cells. Elevated mitochondrial FAO is required for cell growth and survival in response of radiation therapy. Of note, CPT1A/CPT2 expression was also elevated in human recurrent breast cancer tissues compared to biopsy tumors. CRISPR/Cas9 mediated deficiency of CPT1A/CPT2 efficiently enhanced sensitivity of RBCs and RD-BCSCs to radiation treatment. Finally, we provided evidence that mitochondrial FAO likely functions through the ERK signaling pathway to confer resistance to radiation therapy in RBCs.

Our proteomics data demonstrate a different adaptive scale of RD-BCSCs vs. wild type MCF7 cells with a higher mitochondrial protein number and density in RD-BCSCs than MCF7 cells. Such differential stress response may apply to the varied response to therapeutic radiation in primary and recurrent/metastatic breast cancers. The plasticity of human cancer cells and the genetic-independent acquisition of therapeutic resistance may be tightly associated with metabolic reprogramming. CSCs are capable of adjusting their unique metabolic plasticity in order to respond in a timely manner and adapt to hostile environments (46). The current study revealed that enhanced FAO could be a critical step for therapy-resistant cancer cells, especially cancer stem cells, to have a survival advantage, thus they could be used for identifying and developing effective metabolic targets. Altered metabolism is served as one of the hallmarks of cancer and has also been observed in CSCs (47, 48). CSCs have been identified in many types of solid tumors and often result in recurrence and both chemo- and radioresistant in tumors because of their self-renewal and tumorigenic properties (49–51). It has been shown that blocking thioredoxin- and glutathione-dependent metabolism can enhance radiation response of BCSCs (52), indicating that in addition to FAO enhancement, other metabolites could be critical for the survival advantage of CSCs. Accordingly, metabolic adjustment in the CSC populations under different therapeutic modalities should be further investigated.

The cellular energy shift may require a different signaling network to drive cancer cell proliferation and radioresistance. Our current data also reveal a potential crosstalk between CPT1A/CPT2-mediated lipid metabolism and ERK1/2-controlled cell proliferation (Figure 7), implicating a cooperative network of mitochondrial FAO in response to radiation in resistant cancer cells. Although currently there is no direct evidence supporting mitochondrial FAO-mediated ERK1/2 activation, the enhanced mitochondrial products including ATP from TCA cycle may increase the MEK-ERK1/2 cascade. High ATP concentration is indicated in the tumor microenvironment that can activate P2Y2 receptors to enhance BC cell migration through the activation of a MEK-ERK1/2 pathway (53). In addition, mitochondrial OXPHOS can be enhanced by Cyclin B1/CDK1 that can be relocated into mitochondria by radiation leading to phosphorylation of SIRT3, a key keeper for mitochondria homeostasis at the Thr150/Ser159 (54). Furthermore, we have recently observed that mitochondrial homeostasis is enhanced by SIRT3-regulated CTP2 activity in normal mouse liver cells via FAO (unpublished data). Thus, the CPT1A/CPT2-mediated FAO activity may be differently regulated in normal and cancer cells, which warrants further studies.

In summary, this study reveals a previously unknown feature of reprogramming mitochondrial FAO in RBCs due to enhanced CPT1A/CPT2. Thus, targeting CPT1A/CPT2 as well as other mitochondrial FAO elements may serve as a metabolic target to enhance the efficacy of breast cancer radiotherapy.

## Data Availability Statement

All datasets generated for this study are included in the article/Supplementary Material.

## Author Contributions

JL, JJL, and SH designed the study, wrote the manuscript, and were involved in revising the manuscript. SH, RW, and MF performed experiments with the help of NJ, BX. LZ, XZ, AB, MC, JH, and AV helped to analyze the data, prepare and revise the manuscript. H-WC provided the instruction for CRISPR technology. WM and YW generated and analyzed the proteomics data. All authors have approved the final version.

### Conflict of Interest

The authors declare that the research was conducted in the absence of any commercial or financial relationships that could be construed as a potential conflict of interest.

## Supplementary Material

Figure S1 **(A)** Schematic representation of the process for generating RBCs and RD-BCSCs. **(B)** Functional clustering of proteomics of MCF7 and RD-BCSCs via DAVID bioinformatics indicating the protein numbers in categories inducing lipid metabolism, oxidation reduction, DNA repair, stress response, glycolysis, and ATP synthesis in MCF7 cells with or without IR. The intensities of proteins in lipid metabolism were shown in right. **(C)** Repeated proteomics analysis with RD-BCSCs cells, note a group of key FAO enzymes including CPT1A and CPT2 were enhanced by radiation (marked in red in the right).

Figure S2 **(A)** FAO measured in MCF7 and RD-BCSCs cells with or without 5 Gy IR treatment for 24 h. RD-BCSCs treated with 40 μM Etomoxir (ETX) were used as FAO inhibition control, and RD-BCSCs cells treated with 1 μM FCCP as an FAO enhancement control. **(B)** Radiation-induced apoptotic cell death measured by Annexin-V/PI flow cytometry in RD-BCSCs cells, RD-BCSCs treated with 5 Gy IR, ETX (200 μM, 48 h) or combined. **(C)** Repeated experiment in **(B)** using RD-BCSCs CPT1A KO and RD-BCSCs CPT2 KO cells.

Figure S3 **(A)** Mitochondrial fractions were prepared from MCF7 and RD-BCSCs cells treated with or without 5 Gy IR treatment and analyzed by LC-MS and MS/MS on a Q Exactive Plus mass spectrometer. Numbers of proteins detected comparing MCF7 and RD-BCSCs before and after IR are shown on the left. Percentage of enhanced quantitation of the increased protein numbers by radiation are shown in the pie on the right. **(B)** The intensities of mitochondrial protein expression of MCF7 and RD-BCSCs treated with –/+ IR are shown.

Figure S4 **(A)** Functional clustering of mitochondrial proteins via DAVID bioinformatics show the relatively high enhancement of protein numbers in lipid metabolism, oxidation reduction and ATP synthesis in the mitochondria of irradiated RD-BCSCs. **(B)** The two key enzymes in mitochondrial FAO metabolism, CPT1A, and CPT2 (in red), were enhanced by IR in RD-BCSCs.

Table S1 The cluster of proteins involved in fatty acid metabolism enhanced by radiation in RD-BCSCs. The mitochondrial proteomics data were generated with mitochondrial proteins isolated from RD-BCSCs treated with or without IR, followed by digestion with trypsin and analyses by LC-MS and MS/MS on a Q Exactive Plus mass spectrometer. The 17 listed proteins were detected in the proteomic analysis of mitochondrial proteins of RD-BCSCs and classified by UniProtein under the category Lipid Metabolism. The gene symbols, descriptions, and comparison with or without 5 Gy IR are shown in the table, and the CPT1A/CPT2 are marked with yellow.
