# Supplementary figures and images for: CPT1A/2-Mediated FAO Enhancement—A Metabolic Target in Radioresistant Breast Cancer
Source: Front Oncol. 2019 Nov 15;9:1201. doi: 10.3389/fonc.2019.01201 (PMC6873486; doi:10.3389/fonc.2019.01201)

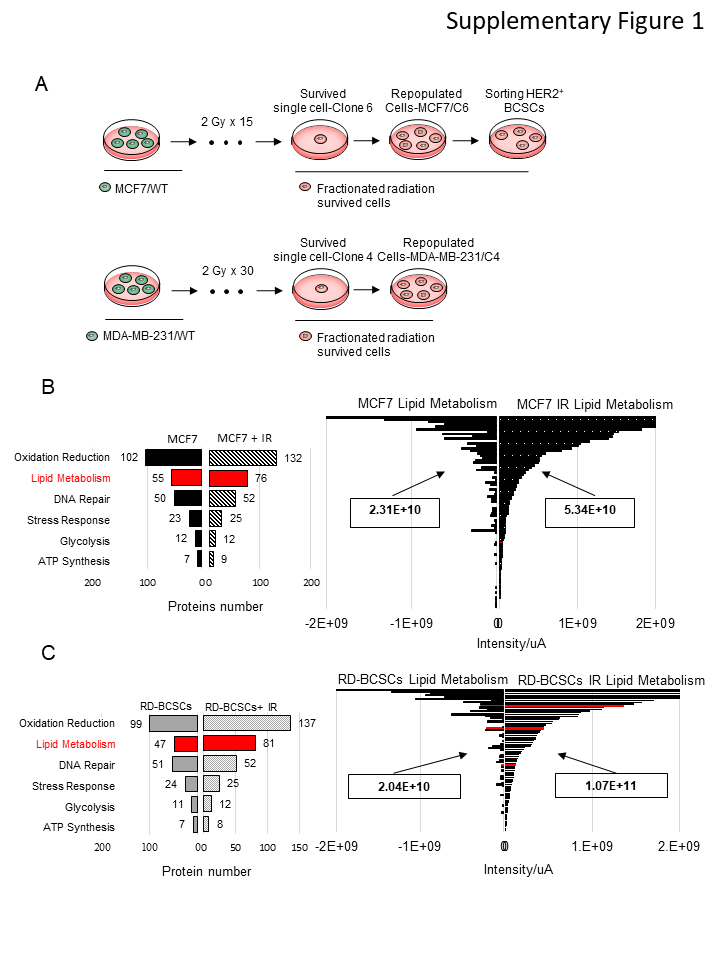

Supplement: Figure S1 — (A) Schematic representation of the process for generating RBCs and RD-BCSCs. (B) Functional clustering of proteomics of MCF7 and RD-BCSCs via DAVID bioinformatics indicating the protein numbers in categories inducing lipid metabolism, oxidation reduction, DNA repair, stress response, glycolysis, and ATP synthesis in MCF7 cells with or without IR. The intensities of proteins in lipid metabolism were shown in right. (C) Repeated proteomics analysis with RD-BCSCs cells, note a group of key FAO enzymes including CPT1A and CPT2 were enhanced by radiation (marked in red in the right). [file Image_1.TIF]

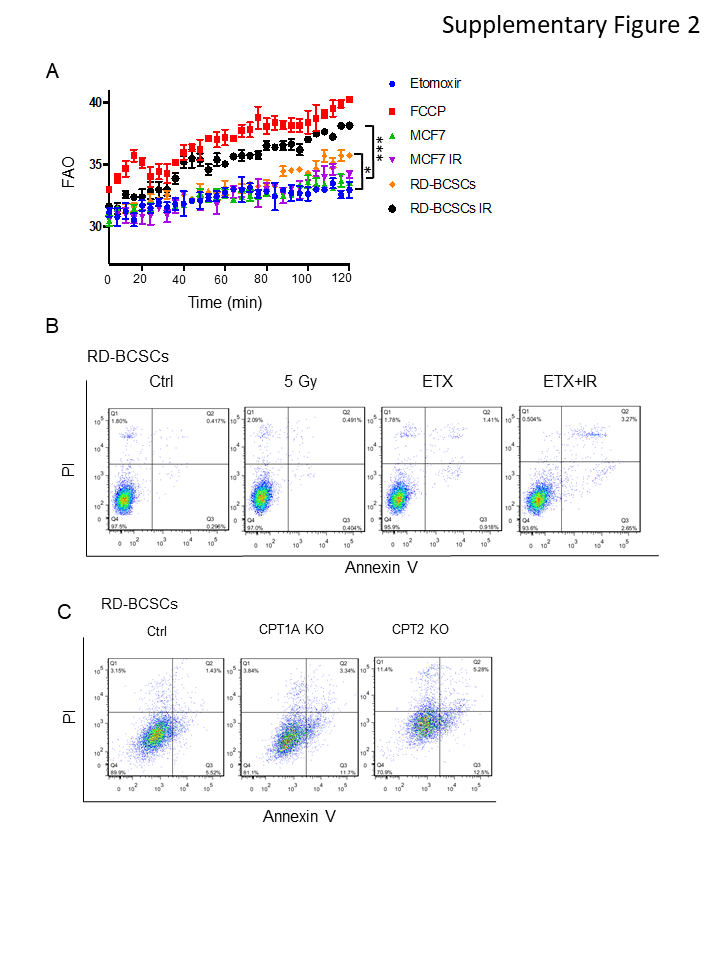

Supplement: Figure S2 — (A) FAO measured in MCF7 and RD-BCSCs cells with or without 5 Gy IR treatment for 24 h. RD-BCSCs treated with 40 μM Etomoxir (ETX) were used as FAO inhibition control, and RD-BCSCs cells treated with 1 μM FCCP as an FAO enhancement control. (B) Radiation-induced apoptotic cell death measured by Annexin-V/PI flow cytometry in RD-BCSCs cells, RD-BCSCs treated with 5 Gy IR, ETX (200 μM, 48 h) or combined. (C) Repeated experiment in (B) using RD-BCSCs CPT1A KO and RD-BCSCs CPT2 KO cells. [file Image_2.TIF]

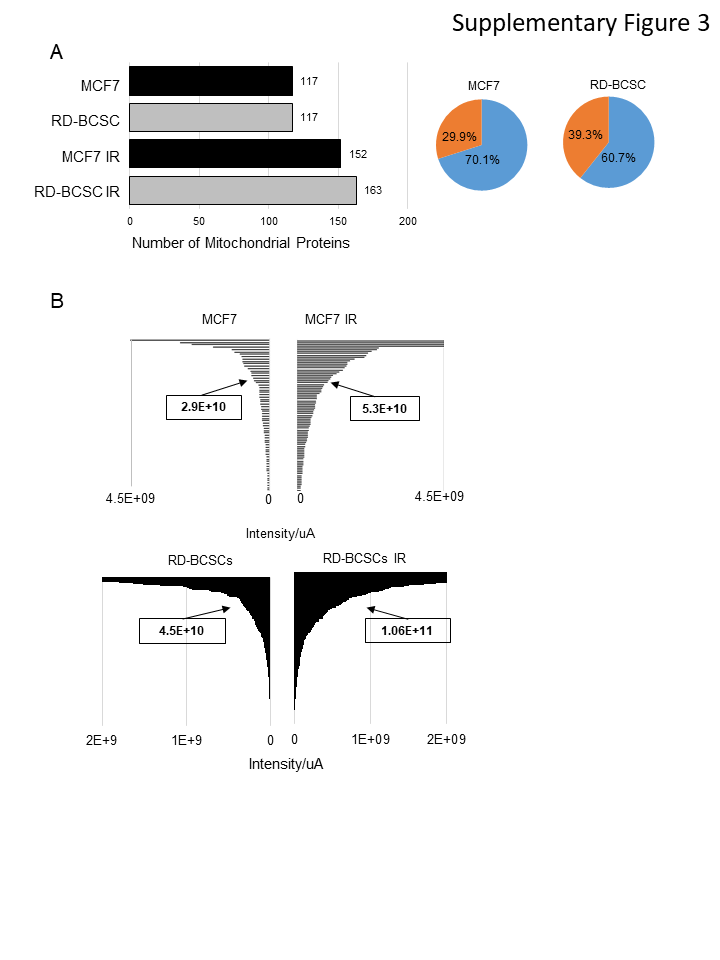

Supplement: Figure S3 — (A) Mitochondrial fractions were prepared from MCF7 and RD-BCSCs cells treated with or without 5 Gy IR treatment and analyzed by LC-MS and MS/MS on a Q Exactive Plus mass spectrometer. Numbers of proteins detected comparing MCF7 and RD-BCSCs before and after IR are shown on the left. Percentage of enhanced quantitation of the increased protein numbers by radiation are shown in the pie on the right. (B) The intensities of mitochondrial protein expression of MCF7 and RD-BCSCs treated with –/+ IR are shown. [file Image_3.TIF]

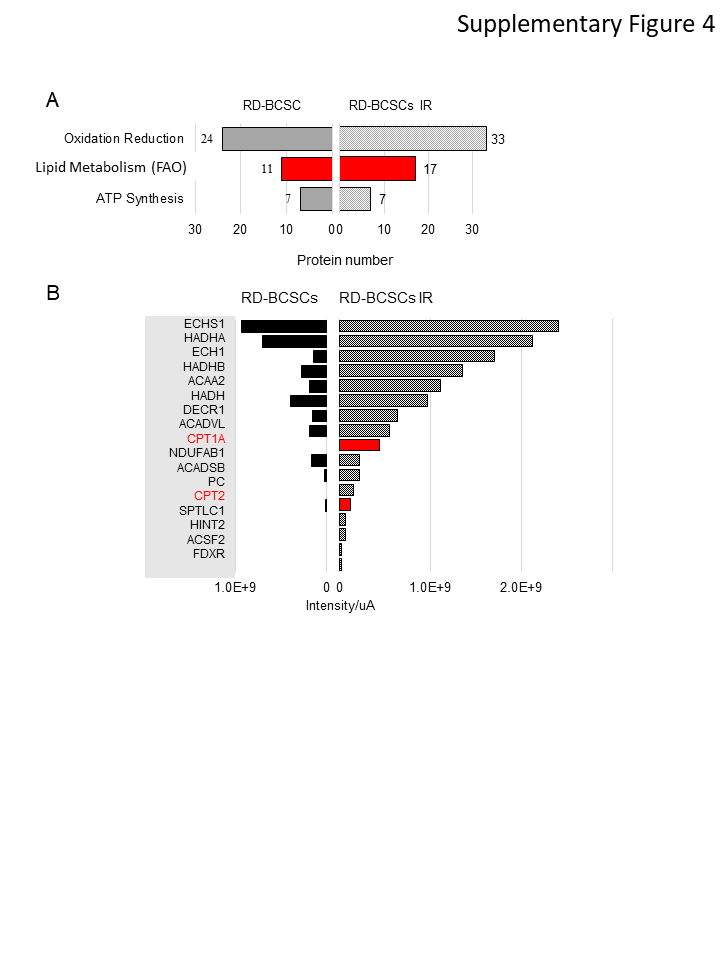

Supplement: Figure S4 — (A) Functional clustering of mitochondrial proteins via DAVID bioinformatics show the relatively high enhancement of protein numbers in lipid metabolism, oxidation reduction and ATP synthesis in the mitochondria of irradiated RD-BCSCs. (B) The two key enzymes in mitochondrial FAO metabolism, CPT1A, and CPT2 (in red), were enhanced by IR in RD-BCSCs. [file Image_4.TIF]

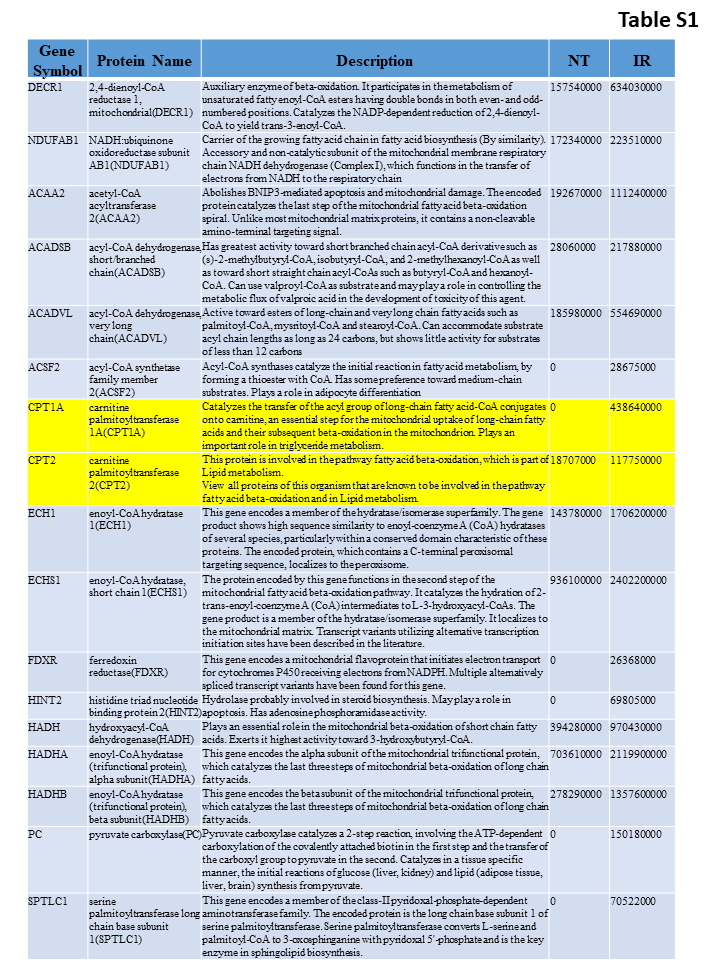

Supplement: Table S1 — The cluster of proteins involved in fatty acid metabolism enhanced by radiation in RD-BCSCs. The mitochondrial proteomics data were generated with mitochondrial proteins isolated from RD-BCSCs treated with or without IR, followed by digestion with trypsin and analyses by LC-MS and MS/MS on a Q Exactive Plus mass spectrometer. The 17 listed proteins were detected in the proteomic analysis of mitochondrial proteins of RD-BCSCs and classified by UniProtein under the category Lipid Metabolism. The gene symbols, descriptions, and comparison with or without 5 Gy IR are shown in the table, and the CPT1A/CPT2 are marked with yellow. [file Data_Sheet_1.zip › Table S1.TIF]
